# Unusual Compression of the Left Internal Mammary Artery Associated with Left Arm Hyperabduction

**Published:** 2019-01

**Authors:** Murat Akcay

**Affiliations:** *Department of Cardiology, Faculty of Medicine, Ondokuz Mayıs University, Samsun, * *Turkey.*

**Keywords:** *Chest pain*, *Mammary artery*, *Arm*, *Coronary angiography*

A 42-year-old male patient presented with a typical chest pain of 2 hours’ duration. The physical examination had no additional features. The electrocardiography showed ST-segment depression in V_2_-V_6_ derivations. There were no additional risk factors except for smoking and family history. The level of cardiac troponin I was high (2.04 ng/mL, normal range between 0 and 0.1 ng/mL). With a diagnosis of non–ST-segment elevation myocardial infarction, a coronary angiography was performed. It showed a severe stenosis in the distal left main, proximal intermediate, anterior descending, circumflex, and middle right coronary arteries (Video 1). Coronary artery bypass surgery was recommended. Left ventriculography showed good ventricular functions. The left internal mammary artery (LIMA) was evaluated with angiography for use as a bypass graft. Nevertheless, the LIMA could not be visualized. As a result, the patient’s left arm was tightened with a blood-pressure cuff and hyperabducted so that the LIMA flow would be better visualized. An atherosclerotic plaque was detected in the proximal LIMA. The hyperabduction of the left arm, however, led to an interruption in the LIMA flow (Figure 1 & Video 2). The compression on the LIMA was resolved after the left arm hyperabduction was corrected (Figure 2 & Video 3). The compression recurred when the left arm was once again hyperabducted, and it was not resolved with nitrate. Chest X-ray did not reveal any accessory rib (Figure 3). A decision was made to perform bypass surgery given the compression on the LIMA. ***The patient underwent a 4-vessel*** coronary artery bypass operation, involving saphenous vein grafts from the aorta to the right coronary artery, the circumflex artery, and the intermediate artery and a LIMA graft to the left anterior descending artery. During the operation, the LIMA flow was good and the LIMA was anastomosed to the left anterior descending artery. The LIMA was released under the pectoralis minor muscle, and the left arm hyperabduction-associated compression was resolved. After the operation, the patient was discharged from the hospital without complications. He was asymptomatic at 6 months’ follow-up, during which the emphasis was upon symptoms related to the hyperabduction of the left arm. There were no symptoms, and nor were there any signs of ischemia in the Stress myocardial perfusion scintigraphy.

The thoracic outlet syndrome has compression-related symptoms on the brachial plexus and the subclavian vessels. Nervous and venous compressions are frequent, and subclavian arterial compression and its related symptoms might be rarely seen.^1^ Compression is frequently caused by the cervical rib, between the scalene muscles; it can also be between the coracoid process and the pectoralis minor muscle, which is known as “the pectoralis minor syndrome” and is often associated with pressure on the subclavian artery.^1^^-^^3^ The pressure and the related symptoms are increased when the left arm is hyperabducted. Chronic compression and trauma lead to endothelial damage and stenosis. The treatment is pectoralis minor tenotomy.^1^^-^^3^ Our case was asymptomatic and incidentally detected during LIMA imaging for bypass surgery. The imaging of the LIMA flow and course is important before bypass surgery.

**Figure 1:**
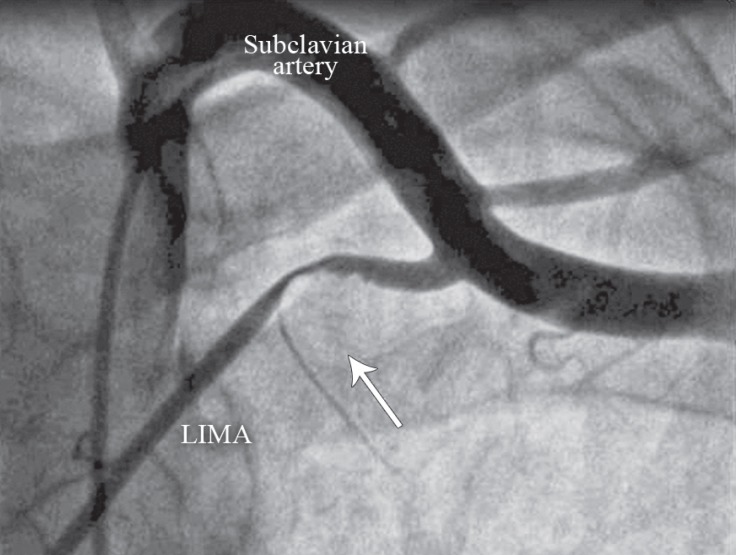
Anteroposterior coronary angiographic view of the subclavian artery and left internal mammary artery (LIMA), showing the proximal LIMA compression and severe flow interruption associated with the hyperabduction of left arm (The arrow indicates the area of the compression and the flow interruption.)

**Figure 2:**
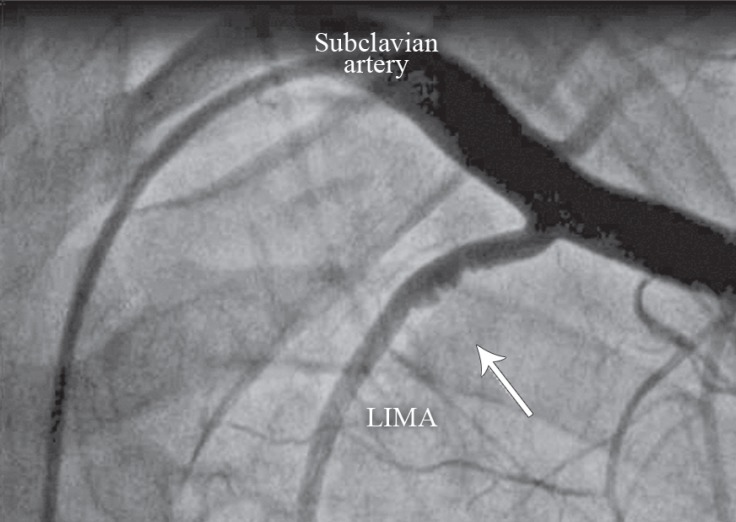
Anteroposterior coronary angiographic view, showing an improved left internal mammary artery flow and compression in the absence of the hyperabduction of the left arm (The arrow indicates improvement in the flow interruption and the arterial wall irregularity.)

**Figure 3:**
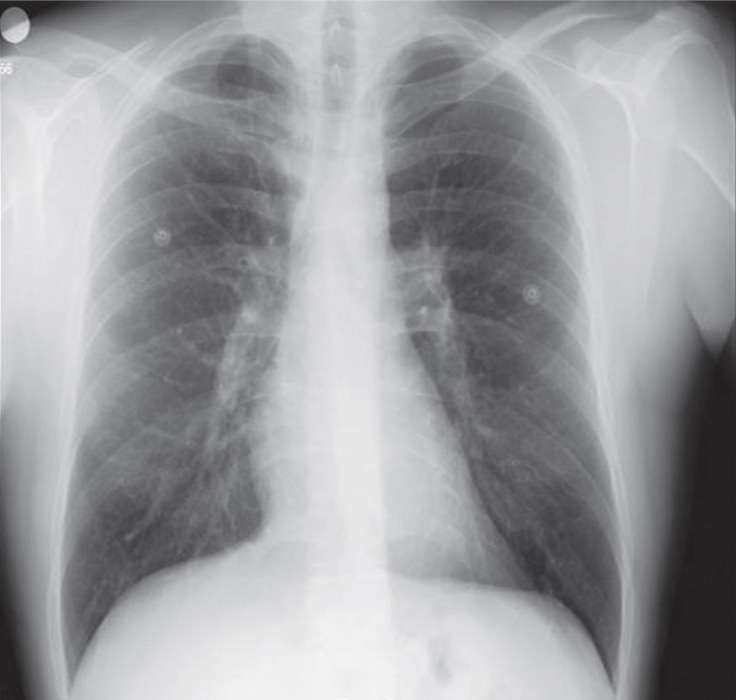
Chest X-ray, showing no accessory cervical rib

***To watch the following videos, please refer to the relevant URLs:***

http://jthc.tums.ac.ir/index.php/jthc/article/view/944/818

Video 1. Angiographic imaging of the coronary vessels

http://jthc.tums.ac.ir/index.php/jthc/article/view/944/819

Video 2. Angiographic imaging of the severe flow interruption in the left internal mammary artery associated with left arm hyperabduction

http://jthc.tums.ac.ir/index.php/jthc/article/view/944/820

Video 3. Angiographic imaging of the improved flow and compression in the left internal mammary artery

## Notes:

***This paper should be cited as: ***Akcay M. Unusual Compression of the Left Internal Mammary Artery Associated with Left Arm Hyperabduction. J Teh Univ Heart Ctr 2019;14(1):40-41.
